# Is kidney function associated with cognition and mood in late life? The Screening for CKD among Older People across Europe (SCOPE) study

**DOI:** 10.1186/s12877-020-01707-4

**Published:** 2020-10-02

**Authors:** Lisanne Tap, Andrea Corsonello, Francesc Formiga, Rafael Moreno-Gonzalez, Johan Ärnlöv, Axel C. Carlsson, Regina Roller-Wirnsberger, Gerhard Wirnsberger, Gijsbertus Ziere, Ellen Freiberger, Cornel Sieber, Tomasz Kostka, Agnieszka Guligowska, Pedro Gil, Sara Lainez Martinez, Rada Artzi-Medvedik, Ilan Yehoshua, Paolo Fabbietti, Fabrizia Lattanzio, Francesco Mattace-Raso, on behalf of SCOPE investigators, Andrea Corsonello, Silvia Bustacchini, Silvia Bolognini, Paola D’Ascoli, Raffaella Moresi, Giuseppina Di Stefano, Cinzia Giammarchi, Anna Rita Bonfigli, Roberta Galeazzi, Federica Lenci, Stefano Della Bella, Enrico Bordoni, Mauro Provinciali, Robertina Giacconi, Cinzia Giuli, Demetrio Postacchini, Sabrina Garasto, Annalisa Cozza, Francesco Guarasci, Sonia D’Alia, Romano Firmani, Moreno Nacciariti, Mirko Di Rosa

**Affiliations:** 1 Department of Internal Medicine, Section of Geriatric Medicine, Erasmus MC, University Medical Center Rotterdam, Rotterdam, The Netherlands; 2 Italian National Research Center on Aging (IRCCS INRCA), Ancona, Fermo and Cosenza Italy; 3 Geriatric Unit, Internal Medicine Department, Bellvitge University Hospital-IDIBELL-L’Hospitalet de Llobregat, Barcelona, Spain; 4 Department of Medical Sciences, Uppsala University, Uppsala, Sweden; 5 School of Health and Social Studies, Dalarna University, Falun, Sweden; 6 Division of Family Medicine, Department of Neurobiology, Care Sciences and Society, Karolinska Institutet, Stockholm, Sweden; 7 Department of Internal Medicine, Medical University of Graz, Graz, Austria; 8 Department of Internal Medicine-Geriatrics, Institute for Biomedicine of Aging (IBA), Friedrich-Alexander Universität Erlangen-Nürnberg, Erlangen, Germany; 9 Department of Geriatrics, Healthy Ageing Research Centre, Medical University of Lodz, Lodz, Poland; 10 Geriatric Department, Hospital Clinico San Carlos, Madrid, Spain; 11 The Recanati School for Community Health Professions at the faculty of Health Sciences, Ben-Gurion University of the Negev, Beer-sheva, Israel; 12 Maccabi Health Organization, Negev district, Tel Aviv-Yafo, Israel; 13 Laboratory of Geriatric Pharmacoepidemiology and Biostatistics, IRCCS INRCA, Via S. Margherita 5, 60124 Ancona, Italy

**Keywords:** Chronic kidney disease, Estimated glomerular filtration rate, Cognition, Cognitive impairment, Mood, Depressive symptoms, Older persons

## Abstract

**Background:**

Chronic kidney disease (CKD), cognitive impairment and depression share common risk factors. Previous studies did not investigate the possible association between kidney function and cognitive and mood disorders in older persons in a broad range of kidney function. The present study explored associations between kidney function, cognition and mood in outpatients of 75 years and over.

**Methods:**

Baseline data of 2252 participants of the SCOPE study, an international multicenter cohort observational study,were used in which community-dwelling persons of 75 years and over were enrolled to screen for CKD Kidney function was estimated with the BIS1-eGFR equation, cognition was assessed with the Mini-Mental State Examination (MMSE) and mood with the Geriatric Depression Scale 15 items (GDS-15). Characteristics were compared across stages of CKD. Mean eGFR values were also compared across categories of MMSE (< 24, 24–26, ≥27) and between groups with high and low score on the GDS-15 (> 5/≤5).

**Results:**

In total, 63% of the population had an eGFR < 60 mL/min. In advanced stages of CKD, participants were older and more often men than in earlier stages (*p* < 0.001). Cardiovascular diseases and diabetes mellitus were more often found in those in advanced stages of CKD (*p* < 0.001), and also cumulative comorbidity scores were higher than in those in earlier stages (*p* < 0.001). Median MMSE was 29 in CKD stage 1–2 and 3, and 30 in CKD stage 4, whereas median GDS-15 score was 2 in all stages of CKD. Mean values of eGFR did not differ across categories of MMSE or between groups with high and low score on the GDS-15. Stratification for albuminuria did not change these results.

**Conclusions:**

Older persons in more advanced stages of CKD did not have lower cognitive scores or higher rates of depressive symptoms than older persons in earlier stages. Future longitudinal studies might give information on the possible effect of kidney function on cognition and mood in late life.

**Trial registration:**

This study was registered prospectively on 25th February 2016 at clinicaltrials.gov (NCT02691546).

## Background

The prevalence of chronic kidney disease (CKD), cognitive impairment and cardiovascular conditions is growing as result of the aging population [1–3]. CKD, cognitive impairment and mood disorders can share common risk factors, such as hypertension and diabetes mellitus [1, 2, 4, 5]. However, whether a decreased kidney function is associated with cognitive and mood disorders in the oldest old is not completely clear.

One of the potential mechanisms underlying the interaction between the kidney and the brain may include the presence of small vessel disease [6]. Persons with CKD have a higher burden of traditional vascular risk factors such as hypertension, diabetes mellitus and dyslipidaemia which are related to small vessel disease in the kidney [6, 7]. The same risk factors are also associated with cerebral white matter lesions, microbleeds, lacunar infarcts and subcortical atrophy which are markers of cerebral small vessel disease increasing the risk of stroke, cognitive decline and dementia [6, 8–11]. Moreover, the presence of small vessel disease may also underlie the association between CKD and depressive symptoms as result of a disruption of brain structures and connecting pathways in mood regulation [12]. Both cognition and mood may also be influenced by metabolic dysregulation and direct effects of lower glomerular filtration rate (GFR) [13–15].

Previous studies suggest that CKD and cognitive impairment might be correlated [16]. However, most studies did not focus on older patients in a broad range of GFR. In addition, in people in all stages of CKD, it was found that the prevalence of depressive symptoms ranged from 7 to 50% and depressive symptoms were more frequent in people in advanced stages of CKD than in people in earlier stages [17]. Nonetheless, most study populations were relatively small and studies did not focus on older persons. Therefore, the relationship between kidney function, cognition and mood in late life remains undetermined.

Since the identification of modifiable risk factors of cognitive and functional decline such as CKD is relevant, this study aimed to investigate the possible association between kidney function, cognition and mood in outpatients aged 75 years and over. We hypothesized that cognitive impairment and depressive symptoms would be more prevalent in advanced stages of CKD than in earlier stages and that persons with cognitive impairment and depressive symptoms would have lower levels of estimated GFR (eGFR) than persons without. Results of this study might provide important information in addition to known risk factors of adverse outcomes in persons with poor kidney function, cognitive impairment and depression. These older persons might then benefit from improved therapeutic strategies when visiting their nephrologist, geriatrician or psychologist.

## Methods

The present study was performed within the framework of the Screening for Chronic Kidney Disease among Older People across Europe (SCOPE) study. The SCOPE study (European Grant Agreement no. 436849), is a multicenter 2-year prospective cohort study involving patients older than 75 years attending outpatient services in participating institutions in Austria, Germany, Israel, Italy, The Netherlands, Poland and Spain. Methods of the SCOPE study have been extensively described elsewhere [18]. The primary objective of the SCOPE study was to investigate the currently available screening methods to identify community-dwelling older patients at risk of kidney disease. Patients with end-stage renal disease or dialysis, a history of solid organ or bone marrow transplantation, an active malignancy or metastatic cancer within 24 months prior to the visit, a life expectancy of less than 6 months, a severe cognitive impairment or patients unwilling to provide consent were ineligible for the SCOPE study. Participants were requested to sign a written informed consent before entering the study. The study protocol was approved by ethics committees at all participating institutions, and complies with the Declaration of Helsinki and Good Clinical Practice Guidelines. Only baseline data are used in the present study*.* Overall, 2461 participants were initially enrolled in the study.

### Kidney function

Serum creatinine (Isotope-Dilution Mass Spectrometry traceable) and albumin-to-creatinine ratio (ACR) were measured at local level by standard methods. Creatinine-based eGFR was calculated in mL/min/1.73m^2^ using the Berlin Initiative Study 1 (BIS1) equation [19]: 3736 × creatinine^-0.87^ × age^-0.95^ × 0.82 (if woman).

The prevalence of stages of CKD was obtained using the Kidney Disease Improving Global Outcomes (KDIGO) guidelines [20]: eGFR ≥60, stage 1–2; 59.9–45, stage 3a; 44.9–30, stage 3b; and 15–30 mL/min/1.73 m^2^, stage 4. Albuminuria was defined as a urine ACR ≥30 mg/g (≥3 mg/mmol) [20].

### Cognition

The cognitive functions were measured by the Mini-Mental State Examination (MMSE) [21]. The MMSE is the most commonly administered psychometric screening tool of cognitive functioning and available and validated in all languages of participating countries. The score ranges from 0 to 30 points, whereas a score < 24 is frequently implemented as the cut-off value for abnormal and indicative of cognitive impairment [22]. A meta-analysis of the accuracy of the MMSE in the detection of dementia and mild cognitive impairment showed a pooled sensitivity and specificity of 71.1 and 95.6% respectively in mixed specialist hospital settings [23]. A MMSE score of < 27 might identify those with a greater risk of cognitive dysfunction, especially in highly educated persons [24]. Therefore, in this study, we defined 3 categories based on the MMSE score: ≥ 27, 24–26 and < 24.

### Mood

The 15-item Geriatric Depression Scale-Short Form (GDS), available and validated in all languages of participating countries, was used to investigate depressive symptoms [25]. It focusses on functional and mood symptoms of depression. The score ranges from 0 to 15 points, with higher scores indicating more depressive symptomatology. The cut-off score most often used for this GDS version is 5 or 6 [26]. A recent systematic review and meta-analysis showed a pooled sensitivity and specificity of 86 and 79%, respectively to detect depression in older persons [27]. In this study, a score > 5 was seen as suggestive of depressive symptoms [25].

### Other variables

Demographic data and socioeconomic status were documented. Information on alcohol use, smoking status, medical history and use of medication was collected and the cumulative illness rating scale for geriatrics (CIRS-G) was calculated in order to score the comorbidity burden by rating the severity of medical problems affecting various organ systems [28]. During the study visit, a comprehensive geriatric assessment (CGA) was performed including also information on basic activities of daily living (ADL) and instrumental ADL (IADL) [29, 30].

### Statistical analysis

Descriptive statistics were expressed as percentage for categorical variables and median and interquartile ranges (IQR) for continuous non-normally distributed variables. First, characteristics were compared across stages of CKD using the Chi square test and Kruskal Wallis test. Second, the correlation between age, MMSE and GDS was explored using the Spearman’s correlation test. Third, mean eGFR values were compared across categories of cognition (MMSE< 24, MMSE 24–26, MMSE≥27) using analysis of variance (ANOVA) in the total population and stratified for the presence of albuminuria. Then, mean eGFR values were also compared between participants with low and high score and GDS (score ≤ 5 and > 5) in the total population and stratified for the presence of albuminuria. A *p*-value of < 0.05 was considered statistically significant.

## Results

Overall, 2461 participants were initially enrolled in the SCOPE study. Of them, 209 participants had missing data on serum creatinine, MMSE and/or GDS leaving a final sample of 2252 participants to be included in the present study. Baseline characteristics are shown in Table 1. Median age was 79.5 years (IQR 77.1–82.9), 55.7% were women and 24.5% were living alone. The majority of the population had hypertension (76.8%), 25.1% had diabetes mellitus, 17.2% had a history of malignancy and 16.6% had congestive heart failure. About 9% had a history of transient ischemic attack and 5.8% have had a stroke. The median total score on the CIRS-G was 8 (IQR 5–11). Only 0.6% of the participants had an eGFR ≥90 mL/min/1.73m^2^, 36.2% were classified as CKD stage 2, 38.3% had CKD stage 3A, 18.6% had CKD stage 3B and 6.3% had CKD stage 4. In 26.7% of the study population, albuminuria was present. Median score on the MMSE was 29 (IQR 27–30), 7.1% of the participants had a MMSE score < 24. Median score on the GDS was 2 (IQR 1–4), 14% of the participants had a GDS score > 5.

**Table 1:** Baseline characteristics (*n* = 2252)

Age, years	79.5 (77.1–82.9)
Women, %	55.7
Living alone, %	24.5
Education, years	11 (8–15)
Smoking status	
Current smoker, %	4.4
Former smoker, %	39.5
Alcohol ≥1 unit a day, %	25.8
BMI, kg/m^2^	27.3 (24.7–30.4)
ADL-independent, %	95.2
iADL-independent, n %	56
MMSE, score	29 (27–30)
MMSE < 24, %	7.1
GDS, score	2 (1–4)
GDS > 5, %	14
Hypertension, %	76.8
Diabetes mellitus, %	25.1
TIA, %	8.7
Stroke, %	5.8
Atrial fibrillation, %	15.3
COPD, %	11.8
Cancer, %	17.2
CHF, %	16.6
Vascular disease, %	12.6
CIRS-G, total score	8 (5–11)
CIRS-G, severity index	1.5 (1.2–1.8)
eGFR-BIS, mL/min/1.73 m^2^	55.6 (45.1–64.4)
90 or more, %	0.6
60–89.9, %	36.2
45–59.9, %	38.3
30–44.9, %	18.6
30 or less, %	6.3
ACR, mg/g	11.2 (33.1–3.4)
Albuminuria, %	26.7

Values are expressed as percentage or median (IQR)

*Abbreviations*: *BMI* Body Mass Index, *(i) ADL* (instrumental) Activities of Daily Living, *MMSE* Mini–Mental State Examination, *GDS* Geriatric Depression Scale, *TIA* Transient Ischemic Attack, *COPD* Chronic Obstructive Pulmonary Disease, *CHF* Congestive Heart Failure, *CIRS-G* Cumulative Illness Rating Scale for Geriatrics, *eGFR-BIS* Estimated Glomerular Filtration Rate, *ACR* Albumin-to-Creatinine Ratio

Characteristics stratified for stages of CKD (stage 1–2 merged) are presented in Table 2. In advanced stages of CKD, participants were older, more often men and former smokers and had fewer years of education than in earlier stages. Comorbidities such as cardiovascular diseases (hypertension, atrial fibrillation, heart failure), diabetes mellitus and history of malignancy were more often found in those in advanced stages of CKD and the CIRS-G total score and severity index were higher than in those in earlier stages. In participants with CKD stage 1–2, 3a and 3b, median MMSE score was 29 (IQR 27–30), whereas median MMSE score in participants with CKD stage 4 was slightly higher with a median value of 30 (IQR 28–30). The proportion of participants with an MMSE score < 24 did not differ across stages of CKD, with a prevalence of 6.4% (stage 1–2), 7.7% (stage 3A), 9.3% (stage 3B) and 5% (stage 4). Median score on the GDS was the same in all stages of CKD, namely 2 (IQR 1–4). A GDS score > 5 was found in 13, 15, 14.1 and 14.2% of the participants in each stage of CKD, respectively. A correlation was found between age and MMSE score (Spearman’s rho − 0.229, *p* < 0.001) and age and GDS score (Spearman’s rho 0.076, *p* < 0.001).

**Table 2:** Baseline characteristics across stages of chronic kidney disease (CKD)

	Stages of CKD				*p*-value
	Stage 1–2 (*n* = 830)	Stage 3A (*n* = 862)	Stage 3B (*n* = 419)	Stage 4 (*n* = 141)	
Age, years	78.6 (76.5–81.1)	79.6 (77.3–83.1)	81 (78.2–85.1)	81 (77.7–84.8)	< 0.001
Women, %	61.1	56.8	47.7	40.4	< 0.001
Living alone, %	23.1	23.7	26	32.6	ns
Education, years	12 (8–16)	11.5 (8–15)	10 (8–13)	8 (8–12)	< 0.001
Smoking status,					
Current smoker, %	5.5	3.6	4.1	3.5	ns
Former smoker, %	35.7	40	44.3	44.9	0.017
Alcohol ≥1 unit a day, %	26.6	24.2	27.2	24.8	ns
BMI, kg/m^2^	26.7 (24.2–29.6)	27.4 (24.8–30.6)	27.7 (25.3–31.0)	27.7 (24.9–31.4)	< 0.001
ADL-independent, %	97.1	95.7	92.1	90.1	< 0.001
iADL-independent, %	66	79.5	72.3	70.2	< 0.001
MMSE, score	29 (27–30)	29 (27–30)	29 (27–30)	30 (28–30)	0.001
MMSE < 24, %	6.4	7.7	9.3	5	ns*
GDS, score	2 (1–4)	2 (1–4)	2 (1–4)	2 (1–4)	ns
GDS > 5, %	13	15	14.1	14.2	ns
Hypertension, %	66.3	77.6	89.3	96.5	< 0.001
Diabetes mellitus, %	19.3	22.6	38.2	36.2	< 0.001
TIA, %	8	8.4	11.2	7.8	ns
Stroke, %	4.3	5.7	7.9	9.2	0.023
Atrial fibrillation, %	9.3	16.6	22	22.7	< 0.001
COPD, %	8.9	11.3	16.7	17.7	< 0.001
Cancer, %	14.2	17.2	20.8	24.8	0.002
CHF, %	8.4	17.9	23.9	34.8	< 0.001
Vascular disease, %	10.8	12.3	16.5	13.5	0.042
CIRS-G, total score	7 (4–10)	7 (5–10)	10 (7–14)	11.5 (8.3–15)	< 0.001
CIRS-G, severity index	1.4 (1.2–1.7)	1.5 (1.2–1.8)	1.7 (1.3–1.9)	1.8 (1.5–2)	< 0.001
eGFR-BIS, mL/min/1.73 m^2^	67.2 (63.6–73.1)	53.8 (50.2–56.9)	39.0 (34.9–42.3)	24 (20.5–27.1)	< 0.001
ACR, mg/g	8.3 (1.9–18.7)	9.4 (3.3–24.3)	24.5 (7–121.8)	161 (53.7–1006)	< 0.001
Albuminuria, %	14	20.8	45.6	81.6	< 0.001

Continuous variables are expressed as median (IQR)

*Abbreviations*: *BMI* Body Mass Index, *(i) ADL* (instrumental) Activities of Daily Living, *MMSE* Mini–Mental State Examination, *GDS* Geriatric Depression Scale, *TIA* Transient Ischemic Attack, *COPD* Chronic Obstructive Pulmonary Disease, *CHF* Congestive Heart Failure, *CIRS-G* Cumulative Illness Rating Scale for Geriatrics, *eGFR-BIS* Estimated Glomerular Filtration Rate, *ACR* Albumin-to-Creatinine Ratio

*Adjusted for age, sex and education

**Fig. 1:**
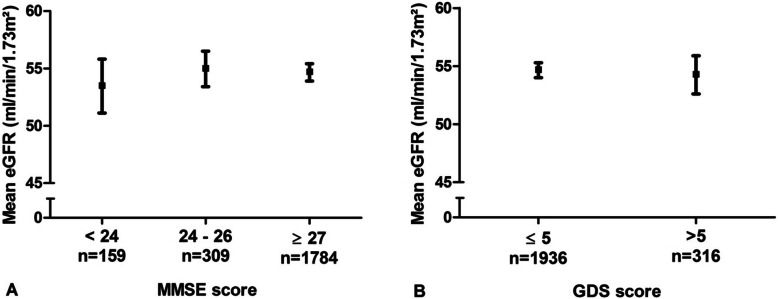
Mean eGFR values across categories of MMSE (**a**) and in participants with low and high score on the GDS (**b**). Notes: Squares represent mean values, bars represent 95% CI. Abbreviations: eGFR, estimated glomerular filtration rate; MMSE, mini-mental state examination; GDS, geriatric depression scale

Figure 1a shows mean values of eGFR across predefined categories of MMSE. One hundred and fifty nine participants had a MMSE score < 24, 309 had a MMSE score of 24–26 and 1784 participants had a MMSE score ≥ 27. Mean eGFR values did not differ across categories; mean values and 95% CI were 53.5 (51.1–55.8), 55 (53.4–56.5) and 54.7 (53.9–55.4) mL/min/1.73m^2^, respectively. Mean values of eGFR did not differ between participants with a low (*n* = 1936) and high score (*n* = 316) on the GDS; mean values and 95% CI were 54.7 (54–55.3) and 54.3 (52.6–55.9) mL/min/1.73m^2^, respectively. Results are shown in Fig. 1b.

**Fig. 2:**
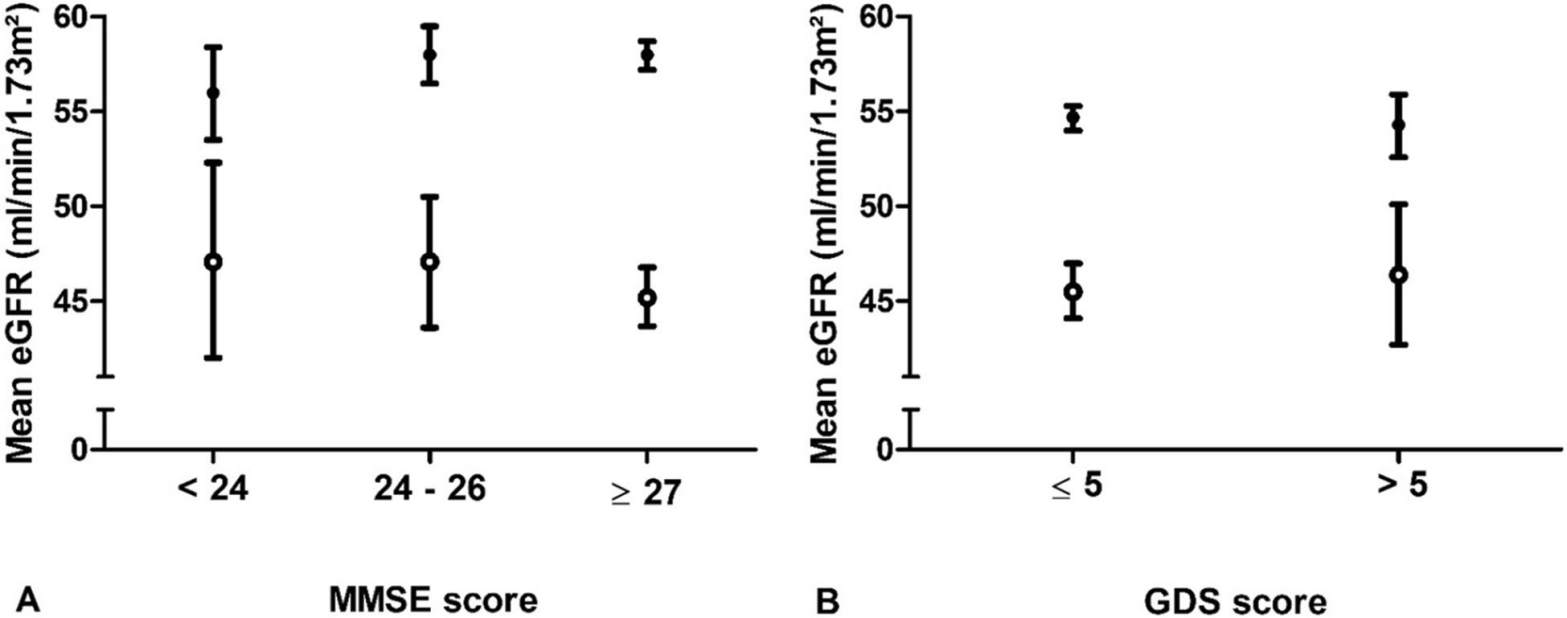
Mean eGFR values across categories of MMSE (**a**) and in participants with low and high score on the GDS (**b**) stratified for albuminuria. Notes: Open dots represent mean values in participants *with* albuminuria, closed dots represent mean values in participants *without* albuminuria, bars represent corresponding 95% CI. *Albuminuria*: *n* = 45, *n* = 86, *n* = 470 from lowest to highest category of MMSE score; participants per category GDS, *n* = 517 (low), *n* = 84 (high). *No albuminuria*: *n* = 114, *n* = 223, *n* = 1314 from lowest to highest category of MMSE score; participants per category GDS, *n* = 1419 (low), *n* = 232 (high). Abbreviations: eGFR, estimated glomerular filtration rate; MMSE, mini-mental state examination; GDS, geriatric depression scale

In Fig. 2a mean values of eGFR in participants with and without albuminuria are shown across categories of MMSE. In participants *with* albuminuria, 45 participants had a MMSE score < 24, 86 had a MMSE score of 24–26 and 470 participants scored ≥27. Mean values of eGFR did not differ across categories; mean values and 95% CI were 47.1 (42–52.3), 47.1 (43.6–50.5) and 45.2 (43.7–46.8) mL/min/1.73m^2^, respectively. In participants *without* albuminuria, the number of participants in the same categories were 114, 223 and 1314, respectively. Mean eGFR values did not differ across categories; mean values and 95% CI were 56 (53.5–58.4), 58 (56.5–59.5) and 58 (57.2–58.7) mL/min/1.73m^2^, respectively. Figure 2b shows mean values of eGFR in participants with and without albuminuria in groups with a low and high score on the GDS. In participants *with* albuminuria, mean values of eGFR did not differ between participants with a low (*n* = 517) and high score (*n* = 84); mean values and 95% CI were 45.5 (44.1–47) and 46.4 (42.7–50.1) mL/min/1.73m^2^. In participants *without* albuminuria, mean values of eGFR did also not differ between groups. Mean eGFR was 58 mL/min/1.73m^2^ (95%CI 57.3–58.7) in participants with a low score (*n* = 1419) and 57.1 mL/min/1.73m^2^ (95% CI 55.4–58.8) in participants with a high score on the GDS (*n* = 232).

## Discussion

Within the framework of the SCOPE study, a large observational cohort including persons aged 75 years and older, we found that early or advanced kidney disease seemed to have no negative influence on cognition or mood in late life. Also, no differences in kidney function were observed in participants with and without cognitive impairment and in those with and without depressive symptoms. The presence of albuminuria did not influence our results.

We found that participants in progressively later stages of CKD were more likely to have risk factors for (cerebral) small vessel disease, such as hypertension and diabetes mellitus, than participants in earlier stages [6]. Also other conditions closely linked to (cerebral) small vessel disease were more prevalent in those in advanced stages of CKD than in those in early stages, such as atrial fibrillation, congestive heart failure and history of stroke [31–33]. The presence of these risk factors is suggested to mediate associations between the kidney and the brain [6], however, we were not able to confirm our prior hypothesis. Previous cross-sectional studies investigated the association between CKD and cognitive impairment in older persons. Most studies investigated this topic in specific populations, such as women with coronary artery disease [34] or only men [35]. In a large sample in the United States of America, it was found that lower levels of kidney function were associated with an increased prevalence of cognitive impairment, assessed with a six-item cognitive screening [36]. In this study, kidney function was assessed by the Modification of Diet in Renal Disease Study (MDRD) equation, an equation that is less reliable than the BIS1 equation in older persons [19], therefore, it is possible that the method used to assess eGFR might have induced bias.

To the best of our knowledge, this is the first cross-sectional study investigating the possible association between kidney function and depressive symptoms in a large study population of community dwelling persons in late life within a broad range of eGFR. In a recent meta-analysis, lower kidney function was associated with higher prevalence of depression [17]. However, only two studies investigated persons with a mean age above 70 years [37, 38]. In a Korean population-based cohort study including almost 1000 participants aged 65 years and older, it was found that an eGFR < 45 mL/min was associated with poor physical quality of life but not with mental health [37]. In older patients admitted with congestive heart failure [38], depression was more prevalent among those with than those without severe CKD (< 30 vs ≥ 30 mL/min). However, since this is a very specific study population, these results cannot be completely compared to our results. Both previous studies used different tools to investigate depressive symptoms [37, 38]: the Short Form 36 (SF-36) health survey and the Beck Depression Inventory (BDI), respectively. Therefore it cannot be excluded that the different methods used might have affected the reported results.

A possible explanation for our findings could lie in the selection of our study population. Eligible persons were community-dwelling, 75 years and older and referred to the outpatient clinics of participating institutions, which might have resulted in a ceiling effect. Almost all participants were under regular medical monitoring at outpatient services, which might have resulted in a better control of cardiovascular risk factors. Therefore, the kidney function and its mediators might no longer be related on the outcomes of interest. One might argue whether kidney function is relevant for the brain (cognition and mood) in the outpatient with multimorbidity in late life or whether all comorbidities in the older outpatient equally contribute to (disturbances in) cognition and mood.

Some limitations of the study need to be discussed. First, the cross sectional design does not allow to investigate causal inferences. Second, persons with relatively good cognition and mood are more likely to volunteer to participate in this study with an extensive protocol, which might have affected our results. Third, persons with end stage renal disease were not included in this study. It might be speculated that mood and cognition are not yet affected in stages 1–4 of CKD due to the asymptomatic nature of the disease. Fourth, there might be a survival bias in which individuals with CKD in advanced stages and possibly cognitive impairment and/or depressive symptoms already died and therefore were not included. Fifth, our study did not include a direct measurement of GFR, however, we used the eGFR-BIS1 equation which is one of the most reliable creatinine-based equations at older age [19]. Eventually, we used the MMSE to assess cognition and the GDS-15 to assess depressive symptoms. We cannot exclude that performing a complete neuropsychological evaluation or using other screening tools might have given different results and might be able to confirm our hypotheses. Ideally, such a complete neuropsychological evaluation can be used in future studies.

This study also has strengths. We have studied a large real-world population of older outpatients in 7 different countries across Europe, therefore our findings can be extrapolated to a large population of European citizens. Second, information on kidney function, cognition and mood were obtained systematically in all participating centers, which makes the results highly reliable.

## Conclusions

In community-dwelling older persons in more advanced stages of CKD, cognitive impairment and depressive symptoms were not more prevalent than in older persons without or in earlier stages of CKD. Kidney function was comparable in those with and without any signs of cognitive or mood disorders. The identification of CKD as modifiable risk factor for cognitive impairment and depressive symptoms in late life might be relevant in order to optimize therapeutic strategies. Longitudinal studies might give additional information on the possible effect of kidney function on mental health in late life. An ongoing prospective SCOPE study is now conducted to investigate effects of CKD progression on these variables.

## Data Availability

The datasets generated and/or analysed during the current study are available in the SCOPE repository (www.scopeproject.eu).

## Abbreviations

ACR: Albumin-to-Creatinin Ratio
ADL: Activities of Daily Living
BDI: Beck Depression Inventory
BIS1: Berlin Initiative Study 1
CGA: Comprehensive Geriatric Assessment
CIRS-G: Cumulative Illness Rating Scale for Geriatrics
CKD: Chronic Kidney Disease
eGFR: Estimated Glomerular Filtration Rate
GDS(− 15): Geriatric Depression Scale (15 items)
GFR: Glomerular Filtration Rate
IADL: Instrumental Activities of Daily living
KDIGO: Kidney Disease Improving Global Outcomes
MDRD: Modification of Diet in Renal Disease Study
MMSE: Mini-Mental State Examination
SCOPE: Screening for CKD among Older People across Europe
SF-36: Short Form 36
